# Molecular structure analyses suggest strategies to therapeutically target SARS-CoV-2

**DOI:** 10.1038/s41467-020-16779-4

**Published:** 2020-06-10

**Authors:** Yi Zhang, Tatiana G. Kutateladze

**Affiliations:** Department of Pharmacology, University of Colorado School of Medicine, Aurora, CO 80045 USA

**Keywords:** Structural biology, Electron microscopy, X-ray crystallography, Diseases

## Abstract

Amid the COVID-19 pandemic, scientists around the globe have been working resolutely to find therapies to treat patients and avert the spreading of the SARS-CoV-2 virus. In this commentary, we highlight some of the latest studies that provide atomic-resolution structural details imperative for the development of vaccines and antiviral therapeutics.

Severe acute respiratory syndrome coronavirus 2 (SARS-CoV-2) has emerged as a novel rapidly spreading human pathogen that causes severe respiratory distress and pneumonia—coronavirus disease 2019 (COVID-19)^1^. This is the third outbreak in recent years caused by emerging coronaviruses after spread of SARS-CoV in 2002 and MERS-CoV in 2012. SARS-CoV-2 and SARS-CoV are closely related and have similar genomes and mechanisms for entry into host cells. The coronavirus spike glycoprotein (S protein), which protrudes from the virion surface, plays a pivotal role in initiating the viral infection facilitating coronavirus attachment to the host cell surface receptor and fusion of viral and host cell membranes.

The spike protein consists of two functional subunits, S_1_ and S_2_ (Fig. 1a), and the receptor-binding domain (RBD) resides within the S_1_ subunit. The RBD of the SARS-CoV and SARS-CoV-2 spike protein binds to the peptidase domain of angiotensin-converting enzyme 2 (ACE2), initiating virus attachment to the host cell surface. The S_2_ subunit mediates virus-host membrane fusion, which requires S protein proteolytic cleavage between the S_1_ and S_2_ subunits and additionally at another, a so called S_2_′ site by host proteases. Owing to its crucial role in viral infection, the spike protein has been a major target for antibody-mediated neutralization of SARS-CoV. Despite high-sequence similarity between the spike proteins of SARS-CoV and SARS-CoV-2, new studies with recovered SARS and COVID-19 patients sera show limited cross-neutralization^2,3^, implying that recovery from one infection may not protect against another^3^, and that the development of vaccines and therapeutics specific to COVID-19 is needed to combat this disease. In that regard, a number of approaches have already been taken and some show promising results. These include the design of new drugs and evaluation of existing therapeutics used individually or in combination, production of antibodies against the SARS-CoV-2 spike protein, particularly its RBD, and targeting other viral and host cell proteins essential for survival and replication of the virus.

**Fig. 1:**
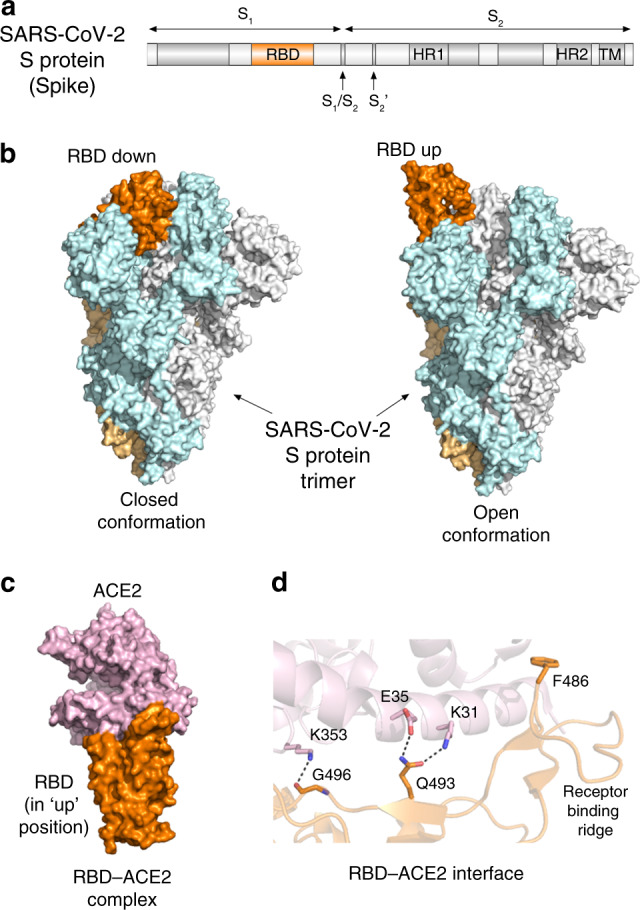
Structural basis for the recognition of human ACE2 by the SARS-CoV-2 spike (S) protein. **a** Domain architecture of the SARS-CoV-2 spike protein. Receptor-binding domain (RBD), heptad repeats (HR1 and HR2), transmembrane domain (TP), and protease cleavage sites S_1_/S_2_ and S_2_′ are labeled. **b** Side views of the spike protein trimer in a closed conformation (left, PDB 6vxx) and open conformation (right, PDB 6vyb). Three protomers are colored light cyan, gray, and light orange. Buried in the closed state RBD (orange) from one of the protomers (light orange) swings up and is ready to bind ACE2 in the open state. **c** Side view of the RBD-ACE2 complex (PDB 6m0j). The RBD position is aligned to that of in (**b**). **d** Zoom in view of the interface of the RBD-ACE2 complex (PDB 6vw1). Dashed lines indicate salt bridges observed in the SARS-CoV-2 complex that are absent in the corresponding SARS-CoV complex.

Recently published structural studies of the SARS-CoV-2 spike protein shed light on the molecular mechanism by which its RBD recognizes human ACE2 and provide an invaluable knowledge in guiding the development of vaccines and inhibitors of viral entry^2,4–7^. Wrapp et al. and Walls et al. report cryo-EM structures of the homotrimeric SARS-CoV-2 spike protein in a prefusion state^2,4^ (Fig. 1b). The data from Walls et al. show that the spike protein exists in two conformations, which is also observed in the other coronaviruses^4^. In the closed conformation, all three RBDs are buried at the interface between three protomers, whereas in the open conformation one of RBDs rotates up and therefore is primed for binding to ACE2. In addition, Wrapp et al. demonstrate that the open conformation represents a predominant state of the spike protein trimer^2^.

How does RBD of the SARS-CoV-2 spike protein binds to the human receptor ACE2? The studies by Shang et al., Lan et al., and Yan et al. illuminate the atomic-resolution details of this interaction^5–7^ (Fig. 1c). Of note, ACE2 normally functions to promote the maturation of angiotensin hormone which controls blood pressure. Aberrant ACE2 levels have been linked to cardiovascular diseases^8^ and may play a role in disease severity observed in COVID-19 patients with comorbid conditions, such as heart, blood, and lung diseases and diabetes. Yan et al. describe a cryo-EM structure of the complex of full length human ACE2 with RBD from the SARS-CoV-2 spike protein and suggest that two spike protein trimers can simultaneously bind to an ACE2 dimer^7^.

The precise binding interface of the SARS-CoV-2 spike RBD and ACE2 can be dissected from the crystal structures of the complex determined by Shang et al. and Lan et al^5,6^ (Fig. 1d). Analyzing the structure, Shang et al. uncovered several hotspots that distinguish binding of SARS-CoV-2 and SARS-CoV^5^. The authors found that overall, the RBD-ACE2 interface in the SARS-CoV-2 complex is larger than the binding interface in the SARS-CoV complex. RBD of SARS-CoV-2 forms more contacts with ACE2, including three additional intermolecular salt bridges, involving Q493 and G496 of RBD and K31, E35, and K353 of ACE2 (dashed lines in Fig. 1d). The receptor-binding ridge of RBD in the SARS-CoV-2 complex adopts a more compact conformation, allowing an adjacent loop to move closer to ACE2 and F486 of RBD to insert in the hydrophobic pocket of ACE2. These structural differences could account for the 4- to 20-fold increase in binding affinity of the SARS-CoV-2 spike RBD toward ACE2 compared to that of SARS-CoV, which was observed independently by several groups^2,4–6^.

Analysis of the crystal structures of RBD from the SARS-CoV spike protein in complex with established SARS-CoV neutralizing human m396 and 80R antibodies reveals that the antibodies are bound in the same binding site as ACE2^9,10^, implying that their neutralizing mechanism is a direct blockage of receptor binding. Lan et al. estimate that 7 out of 21 epitope residues for m396 and 16 out of 25 epitope residues for 80R are not conserved in RBD sequences of SARS-CoV and SARS-CoV-2, which may explain the lack of appreciable cross-reactivity for these antibodies^6^. Interestingly, a recent structure of RBD from the SARS-CoV-2 spike protein bound to another antibody isolated from a SARS patient, CR3022, determined by Yuan et al. shows that CR3022 targets an epitope distal from the ACE2-binding site, which may provide some cross-reactivity that needs to be thoroughly evaluated^11^.

What are other therapeutic targets beyond the receptor-binding domain of the SARS-CoV-2 spike protein? Proteolytic cleavage of the SARS-CoV-2 spike protein by the human serine protease TMPRSS2 is a critical step in the virus entry through the plasma membrane fusion mechanism (Fig. 2), as shown by Hoffmann et al.^12^. The authors demonstrate that camostat mesylate, a clinically proven serine protease inhibitor that halts TMPRSS2 activity, blocks SARS-CoV-2 infection of lung cells^12^. Following binding to ACE2 and disassociation of the ACE2-bound S_1_ subunit, the S_2_ subunit of the spike protein—still attached to the virus envelope through its C-terminal transmembrane domain and additionally cleaved at the S_2_′ site—inserts the cleaved terminus into the host cell plasma membrane and thus physically links both viral and cell membranes. This allows the heptad repeats (HR1 and HR2) of the S_2_ subunit to interact and assemble into a six-helix bundle, which is needed to bring the membranes closer to each other for final fusion. Xia et al. now show that EK1C4, a lipopeptide that targets heptad repeats, is a potent inhibitor of SARS-CoV-2 fusion in cellular assays and in a mouse model^13^. By using human clinical-grade recombinant soluble ACE2, Monteil et al. reduced SARS-CoV-2 viral growth by over 10^3-^fold in Vero cells and suppressed infection in engineered human blood vessel organoids and kidney organoids^14^.

**Fig. 2:**
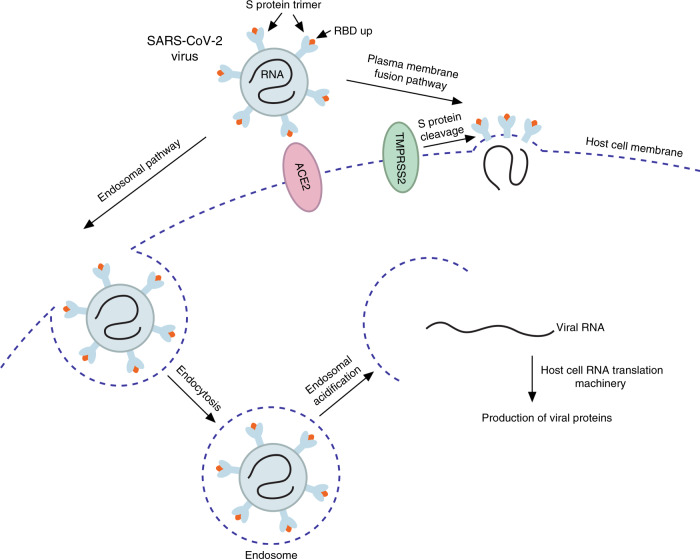
SARS-CoV-2 infection stages and therapeutic targets. A schematic of the SARS-CoV-2 infection stages and current therapeutic targets.

In addition to targeting components of the viral attachment and the membrane fusion machinery, other strategies to eliminate SARS-CoV-2 include impeding virus entry into the host cell through the endosomal pathway and disrupting activities of viral proteins. Ou et al. found that SARS-CoV-2 pseudo-virions enter cells mainly through endocytosis and that inhibition of the proteins essential in the endosomal signaling, such as PIKfyve, TPC2, or cathepsin L by apilimod, tetrandrine, or SID 26681509, respectively, reduces host cell entry^3^. Once inside the host cell, viral RNA uses the host ribosomal machinery to produce important for its own life cycle proteins that also serve as attractive targets for the development of anti-SARS-CoV-2 therapeutics. One of such proteins is the SARS-CoV-2 main protease M^pro^, which is required for processing the polyproteins translated from the viral RNA. Inhibition of the M^pro^ activity impedes viral replication, and the crystal structure of the SARS-CoV-2 M^pro^ in complex with the α-ketoamide inhibitor provides the mechanistic details necessary to advance in this direction^15^.

The speed by which structural information on the SARS-CoV-2 virus has been collected is astonishing. Structural approaches will likely contribute to drug discoveries and validation and aid in the development of vaccines to reduce and eventually eliminate the ongoing public health emergency.
